# Submaximal intensity periods in game-based drills vs. match demands in professional football

**DOI:** 10.3389/fspor.2025.1666652

**Published:** 2025-09-29

**Authors:** Edu Caro, Manuel Lapuente-Sagarra, Toni Caparrós, Miguel Ángel Campos-Vázquez, David Pajón

**Affiliations:** ^1^ National Institute of Physical Education of Catalonia, University of Barcelona, Barcelona, Spain; ^2^ Federación Costarricense de Fútbol (FCRF), San José, Costa Rica; ^3^ FC Barcelona, Barcelona, Spain; ^4^ Sport Research Institute, Autonomous University of Barcelona (UAB), Bellaterra, Spain; ^5^ University Pablo de Olavide, Sevilla, Spain

**Keywords:** submaximal intensity, training load, GPS, football, match demands, small-sided games

## Abstract

**Objective:**

This study aimed to examine the occurrence of submaximal intensity periods (SubMIPs) across several game-based drills according to area per player (ApP) and drill objective, and to compare them with values recorded in competitive matches.

**Methods:**

Fourteen professional football players participated. Data from 1,558 game-based drills events and 247 competitive match records were analyzed using GPS technology. SubMIP events defined as efforts exceeding 85% of each player's 1 min maximal intensity period (MIP) per variable, were calculated for distance, acceleration density (AccDens), high-speed running (HSR), sprints, high metabolic load distance (HMLD), and mean metabolic power (MetPow). Game-based drills were categorized by ApP (<75 m^2^, 75–150 m^2^, >150 m^2^) and objective (possession, four small goals, regular goals).

**Results:**

SubMIP AccDens events were more frequent in game-based drills than in matches, especially in possession drills with smaller ApP. Distance and MetPow events increased with ApP, but none of the game-based drills fully replicated match-level frequencies. HSR, HMLD, and sprint events occurred significantly more often in matches than in any drill.

**Conclusions:**

ApP and drill objective strongly influence physical demands. Although game-based drills do not replicate all match demands, they can be tailored to target specific SubMIP variables. The SubMIP approach provides valuable insights into near-maximal efforts and supports the design of training sessions that optimise player conditioning through repeated high-intensity exposures.

## Introduction

Game-based drills (hereafter “drills”) are widely used in football training since they combine the development of intermittent physical capacities with technical and tactical work and improve player motivation (1–3). In this manuscript, “drills” always denotes game-based drills and does not include isolated exercises (technical, physical, or of any other nature). Studies of these drills can address various aspects, from external and internal loads to the technical and tactical analyses of variables such as the number of completed passes and players’ spatial distribution (4–9).

Analyses of drills tend to focus on two key factors: area per player and drill objective (3, 4, 6, 7, 10, 11). According to previous studies, a larger ApP increases players’ lactate concentration, heart rate, rating of perceived exertion (RPE), total distance covered, frequency of sprints, high-intensity accelerations, high-intensity decelerations, and maximum speed (5, 6, 10, 12–14). By contrast, a smaller ApP increases the frequency of moderate accelerations and decelerations (2–3 m·s^−^^2^) (12, 13).

A change in ApP can also influence the frequency of technical actions such as interceptions, ball controls, dribbles and shots (8–10). The width and length of the drills seem to affect players’ tactical approaches: wider pitches encourage greater use of the wings and more team play, whereas narrower pitches encourage runs in behind the defenders and a more direct style of play (2). In terms of physical demands, wider pitches result in more accelerations and decelerations, whereas narrower pitches require more direct runs and longer distances at high speed (15–17).

Drill objectives also affect players’ physical, technical and tactical responses. Drills with scoring targets at each end of the pitch encourage players to run further at high speed; drills without such targets encourage players to move at a slow or moderate pace (3, 7). An increase in some physical variables has also been noted when smaller goals are used (7). Drills with no scoring target or with a scoring zone led to a higher RPE and higher mean and maximum heart rates (3). The scoring target of a drill can influence a team's tactics. Small goals placed along the end line, midway between the middle of the end line and each corner, increase the number of sideways passes and make the block less compact, whereas if there is only one scoring zone at each end of the pitch, players will be more direct in their play (8).

Studies comparing the physical demands of drills and competitive matches (18–21) show that drills allow players to reach values that are similar to or higher than mean levels in matches for variables such as distance covered and changes of pace (accelerations and decelerations), but not for distance covered at high speed (19, 21).

Most previous studies have compared mean demand in drills and competitive matches. Some studies have focused instead on maximal intensity periods (MIPs)—the periods, during matches or training drills, in which players exert the greatest physical effort. It has been observed that intensity levels are higher when the time windows are shorter (22–24). Comparisons between MIPs in drills and in competitive matches show that, when drills use small spaces, acceleration and deceleration values can be at least as high as during matches, but the values for distance covered, high-speed running (HSR) and sprints are lower (10, 12, 21, 25), except in 10v10 drills with two goalkeepers, in which MIP sprint values exceeded those recorded in matches (25). This shows that, although drills are effective for certain MIP metrics, they are inadequate for others, regardless of the time window used (12, 21, 26). A study on drills without goalkeepers found that the ApP required to replicate the demands of competitive matches over a period of 4 min was 77 m^2^ for accelerations and decelerations, 90 m^2^ for total distance, 187 m^2^ for HSR, and 366 m^2^ for sprints. ApP values in drills with goalkeepers were higher (27).

Recent analyses of MIP's using 1–10 min rolling windows show that true peaks occur in <1% of windows, whereas ≈95% of HSR/sprint and ≈85% of acceleration/deceleration/total-distance windows fall below the peak and vary by position. Thus, while MIPs capture maxima, they under-represent typical exposure for training prescription (28). Consequently, the use of MIPs as the main indicator for designing training drills for team sports has been questioned (18), since, although MIPs identify peak demands, they tend to underestimate the training intensity required to produce specific physiological adaptations, because of their focus on a single maximal intensity event (18, 29) and they ignore other passages of play in which intensity levels, though not at their peak, are still high (30, 31). Alternative indicators have been proposed to address this limitation. Submaximal intensity periods (SubMIPs), for example, allow near-peak demand to be analysed and are therefore better adapted to the intermittent nature of physical output in team sports (31–35). Consequently, threshold-based approaches that quantify exposures exceeding >85% of each player's 1 min peak reveal clear positional and temporal differences. This frequency-based perspective captures repeated near-peak exposures that drive adaptation and justifies using SubMIPs (>85% of the 1 min MIP) as a complement to MIPs (34).

The purpose of this study is: (i) to analyse players’ behaviour in drills using SubMIPs, with results broken down according to the ApP and drill objective; and (ii) to compare the results with those obtained in competitive matches.

## Methods

### Subjects

Fourteen male professional association football players from an Azerbaijan Premier League team took part in this study (weight: 73.74 ± 5.92 kg; height: 1.79 ± 0.05 metres; age: 23.86 ± 3.58 years). Informed consent was obtained in accordance with the Declaration of Helsinki (36) and approval was granted by the Ethics Committee of the Sports Council of Catalonia (number 035/CEICGC/2021).

### Materials

GPS devices were used at all the training sessions and matches analysed in the study (STATSports Apex ProSeries; STATSports, Newry, Northern Ireland). All players used the same device at all times to ensure inter-device reliability (37). The devices had a maximum GPS sampling rate of 18 Hz and included a 600 Hz accelerometer, 10 Hz magnetometer and 400 Hz gyroscope. They weighed 45 g and measured 33 × 80 × 15 mm. Players wore a specially designed vest that held the device in place in the upper back area. The vests and devices had been tested and no significant differences were found between them and other devices that had already produced valid, reliable data for such variables as distance covered and peak speed over distances of 400 m, 128.5 m and 20 m (38). The devices and data were processed by the same duly trained and experienced person.

### Data acquisition and processing

The devices were switched on 15 min before each data-collection session and the app STATSports Apex Live was used to check that they were connected. The data were then segmented according to whether they were collected during drills or competitive matches, then raw data were exported using STATSports software (v3.0.03112) and processed in R (RStudio, Boston, MA, USA). Filtering was applied to the horizontal speed trace to minimise high-frequency noise before numerical differentiation, as recommended to minimise noise without phase distortion and to improve agreement with criterion systems in team-sport GNSS data (39).

Speed signals were low-pass filtered with a 4th-order zero-phase Butterworth (forward–backward, *filtfilt*; fc = 0.75 Hz at 10 Hz sampling; W = 0.15), and instantaneous acceleration (first derivative of filtered speed) was further low-pass filtered with a 1st-order Butterworth (fc = 3.25 Hz; W = 0.65). Zero-phase filtering removes phase distortion; the effective magnitude response equals the square of the single-pass Butterworth (39).

We analysed the following variables: HSR (>19.8 km·h^−^^1^), sprints (>25.2 km·h^−^^1^), acceleration density (AccDens), mean metabolic power (MetPow), distance covered (measured in metres per minute), and high metabolic load distance (HMLD, >25.5 W·kg^−^^1^) (24, 25, 31). An individual reference value (100%) was defined as the mean of the three highest 1 min competition MIPs for each variable (per player) (31, 33, 35). SubMIPs were detected on 60 s rolling windows with a 0.1 s step (10 Hz), Above-threshold windows were merged only when they overlapped (the next window started before the previous one ended). Non-overlapping windows were counted as separate events. Data processing was performed in R (RStudio, Boston, MA, USA), applying a threshold of 85% of each player's individual reference for every variable to get the SubMIP threshold (31, 33). For each training drill, we extracted the number and duration of SubMIP events and stored all outputs in a database for subsequent statistical analyses. To ensure that drills and competitive-match values were comparable, the data were normalised by active duration and reported as event counts (events·min^−^^1^) and exposure time per drill and per match. Files not meeting GNSS quality criteria (≥8 satellites or no excessive dropouts) were excluded (24, 25, 31).

### Competitive matches and drills: characteristics and inclusion criteria

Data were recorded in 15 matches played during the 2019/20 season, in which the team adopted a 5-3-2 formation: five defenders, three midfielders (two defensive, one attacking) and two forwards. Players were included only if they played for at least 45 min per half in at least three halves (31, 33, 40). Based on these criteria, 337 sets of data were obtained on individual player performance, of which 247 were useful for the analysis.

For the drills, a digital odometer was used to measure the length and width of the playing area to calculate the ApP $\left(\frac{\mathrm{Width}\;\times \;\mathrm{Length}}{\mathrm{Number}\;\mathrm{of}\;\mathrm{players}}\right)$. Only data for regular players, and not internal and external floaters, were used in the analysis, and only if the drills were conducted during a competition microcycle, rather than during pre-season training or weeks without a competitive match. In total, 1,612 individual records were obtained from the drills, of which 1,558 were included in the analysis. All training sessions were conducted under the same coaching staff, following a consistent methodology throughout the study period.

**Figure 1:**
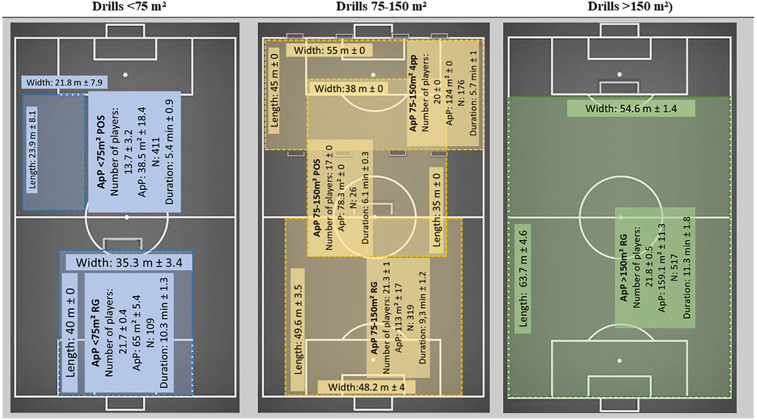
Six game-based drill formats grouped by area per player (ApP) category: <75 m^2^, 75–150 m^2^, >150 m^2^. Panels show mean ± SD for pitch length/width, number of players, ApP, bouts (N), and work-interval duration; dimensions were measured on-field with a digital odometer and ApP = (length × width) ÷ players. Objectives: POS, possession; RG, regular goals; 4SG, four small goals (two per team on each end line).

Drills were classified according to their ApP (< 75 m^2^, 75–150 m^2^ or >150 m^2^) and the type of objective (possession, four small goals per team along each end line (four small goals), or regular goals (5, 7, 11, 41) (Figure 1).

Six different drill formats were used:

• Possession drills with an ApP below 75 m^2^
• Possession drills with an ApP of 75–150 m^2^
• Regular goal drills with an ApP below 75 m^2^
• Regular goal drills with an ApP of 75–150 m^2^
• Regular goal drills with an ApP greater than 150 m^2^
• Four small goal drills with ApP 75–150 m^2^

### Statistical analysis

Analyses were conducted in R (RStudio). For each indicator we reported descriptive statistics of central tendency and dispersion by ApP (m^2^) and drill objective. Outcome distributions were inspected Q–Q plots, and Shapiro–Wilk tests were reported descriptively.

To test differences, we fitted generalised linear models (GLMs; Gaussian family, identity link; *stats::glm*) for each outcome (events·min^−^^1^). We created a single factor condition = ApP × objective (e.g., “<75 m^2^ × possession”, “75–150 m^2^ × four small goals”). The reference level was the competitive match play. Thus, each model coefficient (*β*) represents the mean difference vs. competition on the original outcome scale (events·min^−^^1^ or min·min^−^^1^). For interpretability we also reported an effect size ES_d = *β*/*σ* (*σ* = residual SD from the model), with 95% CIs obtained by dividing the *β* CIs by *σ*. Pairwise comparisons among all conditions were obtained from estimated marginal means using Tukey's adjustment (*emmeans*; two-sided *α* = 0.05; 95% CIs). Model fit indices (AIC, BIC, log-likelihood, deviance, residual degrees of freedom, and residual SD) were recorded (42). Alongside *p*-values, we report standardized effect sizes for model coefficients and pairwise contrasts, computed as d = estimate/*σ* (*σ* = residual SD), with 95% CIs obtained by dividing the corresponding CIs by *σ*.

## Results

### Descriptive results

Distance events did not occur in regular goal drills with ApP < 75 m^2^ and were rare in possession drills with the same ApP. They occurred in all drills with ApP 75–150 m^2^ (highest in four small goals drills), occurred infrequently in regular-goal drills with ApP > 150 m^2^, and occurred more often in competitive matches than in any drill format (Table 1).

**Table 1:** Values are SubMIP events·min^−^^1^ (mean ± SD). N, number of individual player records per condition. Distance, events >85% of the 1 min MIP for total distance (m·min^−^^1^). AccDens, acceleration density (60 s rolling mean of |a|). HSR, high-speed running (>19.8 km·h^−^^1^). Sprint, sprint running (>25.2 km·h^−^^1^). HMLD, distance at metabolic power >25.5 W·kg^−^^1^. MetPow, mean metabolic power. ApP, area per player.

Condition (ApP × Objective)	N	Distance	AccDens	HSR	HMLD	Sprint	MetPow
<75 m^2^ × Regular goals	109	0.000 ± 0.000	0.075 ± 0.097	0.000 ± 0.000	0.000 ± 0.000	0.000 ± 0.000	0.000 ± 0.000
<75 m^2^ × Possession	411	0.000 ± 0.010	0.177 ± 0.163	0.000 ± 0.000	0.000 ± 0.000	0.000 ± 0.000	0.000 ± 0.010
75–150 m^2^ × Regular goals	319	0.001 ± 0.014	0.094 ± 0.108	0.000 ± 0.004	0.000 ± 0.004	0.001 ± 0.007	0.002 ± 0.016
75–150 m^2^ × Possession	26	0.007 ± 0.034	0.143 ± 0.159	0.000 ± 0.000	0.000 ± 0.000	0.000 ± 0.000	0.000 ± 0.000
75–150 m^2^ × Four small goals	176	0.020 ± 0.066	0.075 ± 0.117	0.000 ± 0.000	0.000 ± 0.000	0.000 ± 0.000	0.007 ± 0.033
>150 m^2^ × Regular goals	517	0.010 ± 0.032	0.072 ± 0.084	0.000 ± 0.003	0.000 ± 0.006	0.001 ± 0.007	0.006 ± 0.023
Matches (ApP ≈ 250–350 m^2^)	238	0.042 ± 0.038	0.046 ± 0.034	0.004 ± 0.010	0.006 ± 0.011	0.003 ± 0.008	0.035 ± 0.033

AccDens events occurred in all drill formats. In drills with an ApP of less than 75 m^2^ or 75–150 m^2^, they occurred most frequently in possession drills. In all six drill formats, AccDens events occurred more frequently than in competitive matches (Table 1).

HSR, HMLD and sprint events were practically non-existent in the drills (Table 1).

The drill type in which MetPow events occurred most frequently was in ApP 75–150 m^2^ with four small goals, followed by ApP > 150 m^2^ with regular goals drills, but in both cases they occurred less frequently than in competitive matches.

### Generalized linear models

Generalized linear models revealed significant differences between conditions (ApP category × drill objective) across all performance metrics (Table 2).

**Table 2:** Generalized linear models, indicator (units: events·min^−^^1^). Independent variable was the condition = ApP (m^2^) × drill objective, with Competition as the reference. Each *β* represents the mean difference vs. that reference (on the indicator's scale). ES_d = *β*/*σ* from the model (approximate 95% CIs obtained by dividing the *β* CIs by *σ*). Only terms with *p* < 0.05 are shown (two-sided, no multiplicity adjustment). Effect size reported as Cohen's d (*σ* = residual SD). Magnitude is classified by |d| thresholds: trivial <0.20; small 0.20–0.49; moderate 0.50–0.79; large ≥0.80; the sign of *β* (and d) indicates direction relative to Competition.

Indicador	Condición	*β* (IC95%)	SE	*t*	*p*	Effect Size, d (95% CI)	Magnitude (|d|)
AccDens	<75 m^2^ × RG	0.029 (0.004, 0.055)	0.013	2.260	0.024	0.261 (0.035, 0.488)	Small ↑ vs. Competition
AccDens	<75 m^2^ × POS	0.131 (0.113, 0.149)	0.009	14.329	<0.001	1.167 (1.008, 1.327)	Large ↑ vs. Competition
AccDens	75–150 m^2^ × 4sg	0.029 (0.007, 0.050)	0.011	2.569	0.010	0.255 (0.061, 0.450)	Small ↑ vs. Competition
AccDens	75–150 m^2^ × RG	0.048 (0.029, 0.067)	0.010	4.981	<0.001	0.427 (0.259, 0.594)	Small ↑ vs. Competition
AccDens	75–150 m^2^ × POS	0.097 (0.052, 0.143)	0.023	4.207	<0.001	0.869 (0.464, 1.274)	Large ↑ vs. Competition
AccDens	>150 m^2^ × RG	0.026 (0.009, 0.044)	0.009	2.995	0.003	0.235 (0.081, 0.388)	Small ↑ vs. Competition
Distance	<75 m^2^ × RG	−0.042 (−0.049, −0.035)	0.004	−11.459	<0.001	−1.325 (−1.552, −1.099)	Large ↓ vs. Competition
Distance	<75 m^2^ × POS	−0.041 (−0.046, −0.036)	0.003	−16.081	<0.001	−1.310 (−1.470, −1.150)	Large ↓ vs. Competition
Distance	75–150 m^2^ × 4sg	−0.021 (−0.028, −0.015)	0.003	−6.814	<0.001	−0.677 (−0.872, −0.483)	Moderate ↓ vs. Competition
Distance	75–150 m^2^ × RG	−0.041 (−0.046, −0.035)	0.003	−14.990	<0.001	−1.284 (−1.452, −1.116)	Large ↓ vs. Competition
Distance	75–150 m^2^ × POS	−0.035 (−0.048, −0.022)	0.007	−5.392	<0.001	−1.114 (−1.519, −0.709)	Large ↓ vs. Competition
Distance	>150 m^2^ × RG	−0.032 (−0.037, −0.027)	0.002	−13.028	<0.001	−1.020 (−1.174, −0.867)	Large ↓ vs. Competition
HMLD	<75 m^2^ × RG	−0.006 (−0.007, −0.005)	0.001	−9.378	<0.001	−1.085 (−1.311, −0.858)	Large ↓ vs. Competition
HMLD	<75 m^2^ × POS	−0.006 (−0.0069, −0.0051)	0.000	−13.316	<0.001	−1.085 (−1.244, −0.925)	Large ↓ vs. Competition
HMLD	75–150 m^2^ × 4sg	−0.006 (−0.0071, −0.0049)	0.001	−10.910	<0.001	−1.085 (−1.279, −0.890)	Large ↓ vs. Competition
HMLD	75–150 m^2^ × RG	−0.006 (−0.0067, −0.0048)	0.000	−12.134	<0.001	−1.039 (−1.207, −0.871)	Large ↓ vs. Competition
HMLD	75–150 m^2^ × POS	−0.006 (−0.0083, −0.0038)	0.001	−5.251	<0.001	−1.085 (−1.489, −0.680)	Large ↓ vs. Competition
HMLD	>150 m^2^ × RG	−0.006 (−0.0065, −0.0048)	0.000	−12.972	<0.001	−1.016 (−1.170, −0.863)	Large ↓ vs. Competition
HSR	<75 m^2^ × RG	−0.004 (−0.0054, −0.0034)	0.001	−8.698	<0.001	−1.006 (−1.233, −0.779)	Large ↓ vs. Competition
HSR	<75 m^2^ × POS	−0.004 (−0.0051, −0.0037)	0.000	−12.351	<0.001	−1.006 (−1.166, −0.846)	Large ↓ vs. Competition
HSR	75–150 m^2^ × 4sg	−0.004 (−0.0052, −0.0035)	0.000	−10.119	<0.001	−1.006 (−1.201, −0.811)	Large ↓ vs. Competition
HSR	75–150 m^2^ × RG	−0.004 (−0.0049, −0.0034)	0.000	−11.070	<0.001	−0.948 (−1.116, −0.780)	Large ↓ vs. Competition
HSR	75–150 m^2^ × POS	−0.004 (−0.0061, −0.0026)	0.001	−4.871	<0.001	−1.006 (−1.411, −0.601)	Large ↓ vs Competition
HSR	>150 m^2^ × RG	−0.004 (−0.0049, −0.0036)	0.000	−12.408	<0.001	−0.972 (−1.125, −0.818)	Large ↓ vs. Competition
MetPow	<75 m^2^ × RG	−0.035 (−0.040, −0.030)	0.003	−13.833	<0.001	−1.600 (−1.826, −1.373)	Large ↓ vs. Competition
MetPow	<75 m^2^ × POS	−0.034 (−0.038, −0.031)	0.002	−19.367	<0.001	−1.578 (−1.737, −1.418)	Large ↓ vs. Competition
MetPow	75–150 m^2^ × 4sg	−0.028 (−0.0324, −0.0239)	0.002	−12.988	<0.001	−1.291 (−1.486, −1.096)	Large ↓ vs. Competition
MetPow	75–150 m^2^ × RG	−0.033 (−0.037, −0.0297)	0.002	−17.845	<0.001	−1.528 (−1.696, −1.361)	Large ↓ vs. Competition
MetPow	75–150 m^2^ × POS	−0.035 (−0.0437, −0.0261)	0.005	−7.745	<0.001	−1.600 (−2.005, −1.195)	Large ↓ vs. Competition
MetPow	>150 m^2^ × RG	−0.029 (−0.0326, −0.0259)	0.002	−17.123	<0.001	−1.341 (−1.495, −1.188)	Large ↓ vs. Competition
Sprint	<75 m^2^ × RG	−0.003 (−0.0047, −0.0021)	0.001	−5.074	<0.001	−0.587 (−0.813, −0.360)	Moderate ↓ vs. Competition
Sprint	<75 m^2^ × POS	−0.003 (−0.0043, −0.0025)	0.000	−7.204	<0.001	−0.587 (−0.746, −0.427)	Moderate ↓ vs. Competition
Sprint	75–150 m^2^ × 4sg	−0.003 (−0.0045, −0.0023)	0.001	−5.902	<0.001	−0.587 (−0.782, −0.392)	Moderate ↓ vs. Competition
Sprint	75–150 m^2^ × RG	−0.003 (−0.0038, −0.0019)	0.000	−5.738	<0.001	−0.492 (−0.659, −0.324)	Small ↓ vs. Competition
Sprint	75–150 m^2^ × POS	−0.003 (−0.0057, −0.0011)	0.001	−2.841	0.005	−0.587 (−0.992, −0.182)	Moderate ↓ vs. Competition
Sprint	>150 m^2^ × RG	−0.003 (−0.0037, −0.0019)	0.000	−6.243	<0.001	−0.489 (−0.643, −0.336)	Small ↓ vs. Competition

ApP, area per player (m^2^); RG, regular goals; POS, possession; 4 sg, four small goals; HSR, high-speed running; HMLD, high metabolic load distance; MetPow, metabolic power.

Distance events were more frequent in drills with larger ApP and in competitive matches, and less frequent in possession drills. AccDens events were higher in possession drills and lower in match play. HMLD, HSR and sprint events occurred more frequently during competitive matches than in any drills format.

MetPow events were higher in large-area drills (75–150 m^2^ four small goals; >150 m^2^ regular goals) but remained lower than in competitive matches. Model fit was adequate across outcomes (AIC −14,422 to −2,754; BIC −14,378 to −2,710; log-likelihood 1,385–7,219; deviance 0.034–22.487; residual SD *σ* 0.004–0.112), with AccDens exhibiting the largest residual dispersion (*σ* ≈ 0.112; deviance ≈ 22.49).

See Table 2 for full GLM outputs.

Effect sizes (d with 95% CIs) are reported alongside *p*-values; magnitudes were generally small–to–moderate between drill formats and larger for competition- vs. -drill contrasts (positive for AccDens, negative for distance, HMLD, HSR, sprint, and MetPow).

**Figure 2:**
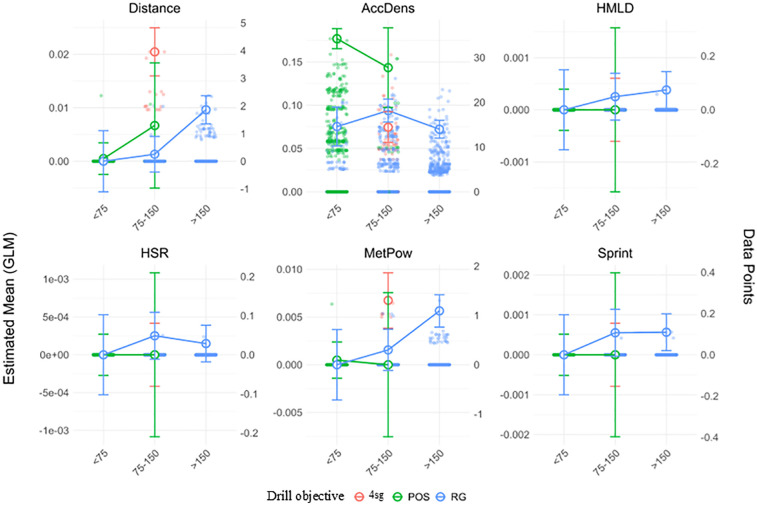
Estimated marginal means (±95% CI) of SubMIP event rates (events·min^−1^) for game-based drills by area per player (ApP: <75, 75–150, >150 m^2^) and drill objective. Lines/markers 4 sg (red), four small goals; POS (green), possession; RG (blue), regular goals. Semi-transparent dots show raw drill observations (jittered). Variables: Distance (m·min^−1^ AccDens, acceleration density; HSR, high-speed running (>19.8 km·h^−1^, Sprint (>25.2 km·h^−1^); HMLD, high metabolic-load distance (>25.5 W·kg^−1^); MetPow, mean metabolic power. Estimates are from GLMs (Gaussian, identity) with condition = ApP × objective; contrasts and CIs obtained via estimated marginal means (emmeans). Competitive match (Comp MD) is not displayed in this figure.

Post hoc comparisons using estimated marginal means revealed significant differences between ApP categories and drill objectives (Figure 2).

For distance events, ApP 75–150 m^2^ with four small goals drills showed higher values than ApP < 75 m^2^ possession, ApP < 75 m^2^, ApP 75–150 m^2^ and ApP > 150 m^2^ with regular goals drills (Δ ≈ 0.019–0.020, *p* < 0.001), and lower values than competitive matches (Δ ≈ 0.021, *p* < 0.001). This suggests that four small goals drills with moderate ApP require greater physical exertion than other drill formats, though still below match demands.

AccDens events were significantly more frequent in ApP < 75 m^2^ possession drills compared to all with regular goals and four small goals formats (Δ ≈ 0.08–0.10, *p* < 0.001), and also more frequent than in competitive matches (Δ ≈ 0.131, *p* < 0.001). Conversely, ApP >150 m^2^ with regular goals drills had lower AccDens than ApP 75–150 m^2^ possession (Δ ≈ 0.07, *p* = 0.027), highlighting the influence of both ApP and objective type.

For HSR, HMLD and sprint events, all values were significantly higher in competitive matches than in any drills format (Δ ≈ 0.0028–0.0060, *p* < 0.001), reinforcing the idea that matches induce more intense locomotor demands than training drills.

**Figure 3:**
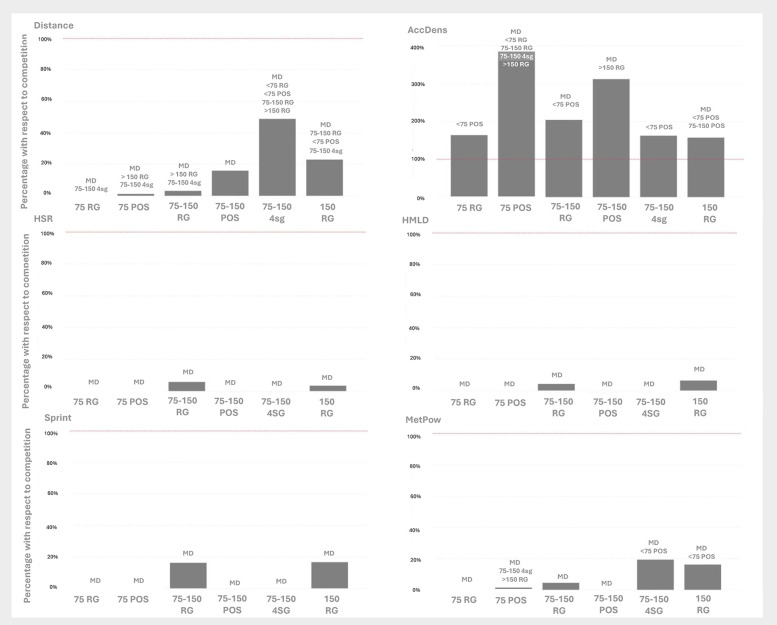
Percentage of SubMIP events (events·min^−1^) in each drill format, expressed relative to competitive match values (MD = 100%). ApP bands: <75 m^2^ (75), 75–150 m^2^ (75–150), >150 m^2^ (150). Drill objectives: POS, possession; 4 sg, four small goals; RG, regular goals (with goalkeepers). Variables: Distance (m·min^−1^), AccDens, acceleration density; HSR, high-speed running (>19.8 km·h^−1^); Sprint (>25.2 km·h^−1^); HMLD, high metabolic-load distance (>25.5 W·kg^−1^); MetPow, mean metabolic power. Bar-top annotations: MD, significantly different from competitive matches (p < .05); the additional labels under “MD” list other drill conditions that differ significantly from the current bar (Tukey-adjusted post-hoc from GLM with Gaussian identity via emmeans).

Finally, MetPow events occurred more frequently in matches (Δ ≈ 0.029–0.035, *p* < 0.001) and were also significantly higher in ApP 75–150 m^2^ with four small goals and >150 m^2^ with regular goals drills than in ApP < 75 m^2^ possession (Δ ≈ 0.005–0.006, *p* < 0.05), suggesting that larger spaces and goal-oriented tasks contribute to greater metabolic loads (Figure 3).

## Discussion

The objectives of this research were to identify differences in the occurrence of SubMIP events in drills by ApP level and drill objective, and to compare the results with the occurrence of such events in competitive matches. Differences in the frequency of SubMIP events suggest that the ApP and the drill objective significantly influence the physical demands on players. These differences are consistent with comparisons between drills and competitive matches, which reveal that some drill formats fail to replicate the demands of competitive matches for some variables, but generate higher values for others, such as AccDens. The results suggest that drills should be refined to better simulate the demands of competitive matches to ensure that players are properly stimulated across all key performance metrics.

The SubMIP approach takes the analysis beyond peak exertion levels. By also covering repeated and accumulated near-peak exertion periods, this approach offers a more accurate picture of sustained performance in the various drill formats. To the best of the authors’ knowledge, no previous study has included in-depth analysis of SubMIP events in several drill formats.

In formats with an ApP below 75 m^2^, HSR, HMLD, sprint and MetPow events were noticeably absent, probably because a small playing area prevents players from reaching high speeds as frequently. In the same formats, SubMIP distance events occurred less frequently than in drills with a higher ApP and in competitive matches, which is consistent with previous studies that analysed mean demand (5, 6, 10, 13, 20) or MIP (21, 25). This finding provides further evidence of the benefits of analysing SubMIP events, since it shows that some near-maximal exertion periods are limited by the space available to players, thus highlighting specific conditions that affect player performance in these variables.

AccDens events, by contrast, occurred most frequently in formats with an ApP below 75 m^2^, especially in possession drills, with frequencies exceeding those observed in competitive matches and all other drill formats except ApP 75–150 m^2^ possession. The higher number of AccDens events in possession drills is consistent with the results of previous studies that compared drills with and without goalkeepers (27, 43).

The higher frequency of SubMIP AccDens events in drills with a smaller ApP could be because the smaller team sizes in such formats encourage players to increase their movement (more off-the-ball movements for the team in possession and more defensive duels) and because players accelerate from a stationary position. In competitive matches, by contrast, such actions are more intense but less frequent. In other words, a smaller ApP increases the frequency of such actions, whereas a larger ApP increases their intensity (13).

Formats with an ApP of 75–150 m^2^ had a greater impact on distance events than formats with a smaller ApP (Figure 3). Although drills with a larger playing area allow greater movement, the space is still too restricted to replicate the demands of competitive matches, as indicated in previous studies (10, 20, 21, 25). In the ApP 75–150 m^2^ with four small goals format in particular, AccDens values were significantly higher than in all other drill formats except in possession with ApP 75–150 m^2^ drills, probably because players had to move constantly to cover their goals and to create space with lateral passes, which encouraged greater width and forced players to cover greater distances (7, 8). These results highlight the advantage of using SubMIP analysis to measure the accumulation of efforts that involve significant sustained exertion despite not representing maximum match-level intensity.

AccDens events were more frequent in ApP 75–150 m^2^ with regular goals and possession drills than in competitive matches, which suggests that drills with an ApP of 75–150 m^2^ frequently force players to change pace. This finding—which held true irrespective of whether the comparison was with competition averages or various MIP time windows—supports the idea that almost any drills format can stimulate players to equal or surpass the changes of pace they make in matches (20, 23, 25).

The results of this study confirm that, for possession drills to stimulate SubMIP AccDens events more than competitive matches, the ApP can be smaller than the level suggested previously based on an analysis of the mean levels of the variables (27). It is important to bear in mind, however, that the two studies were not identical, though they did address similar aspects, including how the ApP affects the changes of pace required of players.

For higher-intensity events, such as HSR, HMLD and sprints, an ApP of 75–150 m^2^ did not replicate the demands of SubMIP periods in competitive matches, which indicates that during matches, players engage in high-speed actions more frequently than during drills (21, 27, 44). The ApP required to replicate those demands is estimated at 166 m^2^ for HSR and 295 m^2^ for sprints when using conventional metrics. These estimates increase to 187 m^2^ for HSR and 366 m^2^ for sprints in the MIP analysis (27, 44) regular goals drills were the only ones in which SubMIP events were recorded for these variables and, though the results were not statistically significant, they add weight to the hypothesis that the type of objective modifies players’ tactical behaviour and the physical demands placed on them (13, 27).

For the MetPow variable, although an ApP of 75–150 m^2^ generated some SubMIP events, they were significantly less frequent than in competitive matches, which suggests that metabolic demands are higher during matches and cannot be fully replicated with an ApP of 75–150 m^2^. The estimated minimum ApP required to replicate match-level per-minute stimuli is 177 m^2^ (27). The only significant difference was found in the ApP 75–150 m^2^ four small goals format drills, in which the values were higher than in ApP < 75 m^2^ possession drills, probably due to players’ unique tactical behaviour in this format, as discussed previously.

Analysis of the results in the drills with an ApP that was greater than 150 m^2^ shows that distance events occurred less frequently than in competitive matches and App 75–150 m^2^ four small goals drills, but more frequently than in regular goals drills with the same ApP (which use the same type of scoring target). This suggests that, when players have more space, they have more freedom to move around and therefore to engage in more SubMIP events, albeit without replicating the values attained during matches (5, 6). This result differs from the results observed in studies based on per-minute averages (44) or in MIP analysis (25), in which match-level values were reached. One possible explanation is that the ApP of these >150 m^2^ with regular goals drills was often below the 187 m^2^ threshold that, according to Ribioli et al. (2020), is necessary for match-level values to be attained. Furthermore, given that the threshold needs to be exceeded for 1 min to generate SubMIP events, the duration of a drill is an important variable, since the analysis is not limited to isolated exertion periods, unlike in previous studies.

The frequency of SubMIP AccDens events in ApP > 150 m^2^ with regular goals drills was significantly lower than in ApP 75–150 m^2^ and < 75 m^2^ possession drills, probably because larger spaces reduce the need for changes of pace and because players’ tactical behaviour changes when the drill objective is different. The effect of the ApP on SubMIP AccDens events is consistent with the inverse relationship between the ApP and moderate accelerations identified in previous studies (12, 13, 21). The values attained in this format exceeded the mean SubMIP AccDens values recorded in competitive matches, which is also consistent with previous analysis of MIP values for changes of pace (12, 23).

High-intensity events such as HSR, HMLD, sprints and MetPow occurred in ApP > 150 m^2^ with regular goals drills less frequently than in competitive matches, but slightly more frequently than in other drills. The additional space allowed players to reach higher speeds (13, 21), but not enough to replicate the SubMIP values of competitive matches. This was consistent with the findings of previous MIP analyses (12, 21). In studies that analysed mean values for these metrics, by contrast, it was shown that drills with a larger ApP could replicate the demands of competitive matches (25).

Finally, the only drills in which SubMIP HSR, HMLD and sprint events occurred—albeit without significant differences—were the two regular goals drill formats with an ApP greater than 75 m^2^. This finding adds weight to the idea that, in regular goals drills, players adopt tactics that encourage HSR (13).

This study has several limitations. First, its analysis of only six drill formats limits the broader applicability of the results. Second, the use of different scoring targets with different pitch sizes may have influenced players’ physical responses. Third, the specifc and small sample size may limit statistical power and generalizability. Finally, the results depend on MIP values that vary from one player to another.

## Conclusions

The frequency of SubMIP events differed significantly among different types of drills and between drills and competitive matches. The ApP and drill objective strongly influence the physical demands on players. Although some drill formats did not fully replicate the demands of competitive matches, AccDens values were similar—and sometimes higher—than the values attained in matches. Furthermore, the SubMIP approach allows detailed monitoring of the repetition and accumulation of near-peak exertion periods, allowing coaches to better optimise training loads.

## Practical applications

The results suggest that drills can be adapted to better address the demands of competitive matches. To stimulate high-intensity events such as HSR and sprints, drills should have an ApP greater than 150 m^2^ and should be supported by specific high-speed exercises. The intensity of certain variables in regular goals drills could be increased to levels near those of competitive matches. Small spaces (ApP < 75 m^2^ per player) lead to more demanding SubMIP AccDens events and require players to engage in changes of pace more frequently, especially in possession drills. Since no single drills format replicates all the demands of competitive matches, coaches should combine different ApP levels and drill objectives to ensure that players are fully stimulated during training sessions. Where feasible, coaches should ensure that players attain near-match SubMIP levels during the training week.

While our study did not track injuries, frequent SubMIP exposures reflect repeated near-maximal demands. When poorly periodized (e.g., abrupt increases across key external-load parameters), such exposures may contribute to maladaptation, potentially affecting readiness and injury risk. Conversely, progressive, individualized dosing of drill formats with higher SubMIP occurrence can better align training with match demands while managing risk.

This methodological proposal allows loads to be adapted to the individual needs of each athlete. SubMIP-based analysis provides an additional tool for the detailed monitoring of physical demands in sports with intermittent loads.

## Perspectives

Researchers should explore a wider range of drill formats, examine alternative temporal windows for SubMIP detection (in addition to the 60 s window) and develop a standardized methodology for analysing SubMIP events. They should also continue to investigate training loads, since the frequency and intensity of effort needed to optimise the adaptation of individual athletes remains unknown.

## Data Availability

The datasets presented in this study can be found in online repositories. The names of the repository/repositories and accession number(s) can be found in the article/Supplementary Material.

## References

[1] Hill-Haas SV Dawson B Impellizzeri FM Coutts AJ. Physiology of small-sided games training in football: a systematic review. Sports Med. (2011) 41(3):199–220. 10.2165/11539740-000000000-00000 21395363

[2] Campos Vázquez MÁ Jiménez Iglesias J. Periodización integral. Un modelo holístico para el futbol actual. In: Periodización Integral. Firts. Torrazza Piemonte: Amazon KDP (Independently published) (2024). p. 159–96.

[3] Praça GM Andrade AGP Bredt SDGT Moura FA Moreira PED. Progression to the target vs. regular rules in soccer small-sided games. Sci Med Football. (2021) 6(1):66–71. 10.1080/24733938.2020.1869811 35236221

[4] Clemente FM Afonso J Castillo D Arcos AL Silva AF Sarmento H. The effects of small-sided soccer games on tactical behavior and collective dynamics: a systematic review. Chaos Soliton Fract. (2020) 134:109710. 10.1016/j.chaos.2020.109710

[5] Casamichana D Castellano J. Time-motion, heart rate, perceptual and motor behaviour demands in small-sides soccer games: effects of pitch size. J Sports Sci. (2010) 28(14):1615–23. 10.1080/02640414.2010.521168 21077005

[6] Praça GM Chagas MH Bredt SDGT Andrade AD. Small-sided soccer games with larger relative areas result in higher physical and physiological responses: a systematic and meta-analytical review. J Hum Kinet. (2022) 81(1):163–76. 10.2478/hukin-2022-0013 35291625 PMC8884881

[7] Sarmento H Clemente FM Harper LD Costa Id Owen A Figueiredo AJ. Small sided games in soccer–a systematic review. Int J Perf Anal Sport. (2018) 18(5):693–749. 10.1080/24748668.2018.1517288

[8] Clemente FM Sarmento H. The effects of small-sided soccer games on technical actions and skills: a systematic review. Hum Mov. (2020) 21(3):100–19. 10.5114/hm.2020.93014

[9] Owen AL Wong DP Mckenna M Dellal A. Heart rate responses and technical comparison between small- vs. large-sided games in elite professional soccer. J Strength Cond Res. (2011) 25(8):2104–10. 10.1519/JSC.0b013e3181f0a8a3 21642858

[10] Clemente FM Praça GM Aquino R Castillo D Raya-González J Rico-González M Effects of pitch size on soccer players’ physiological, physical, technical, and tactical responses during small-sided games: a meta-analytical comparison. Biol Sport. (2023) 40:111–47. 10.5114/biolsport.2023.110748 36636192 PMC9806761

[11] Aguiar M Botelho G Lago C Maças V Sampaio J. A review on the effects of soccer small-sided games. J Hum Kinet. (2012) 33(1):103–13. 10.2478/v10078-012-0049-x 23486554 PMC3588672

[12] Dalen T Samael S Stenens T Hjelde GH Kjosnes T Wilsoff U. Diferences in acceleration and high-intensity activities between small-sided games and peak periods of official matches in elite soccer players. J Strength Cond Res. (2019) 35(7):2018–24. 10.1519/JSC.0000000000003081 30741867

[13] Gaudino P Alberti G Iaia FM. Estimated metabolic and mechanical demands during different small-sided games in elite soccer players. Hum Mov Sci. (2014) 36:123–33. 10.1016/j.humov.2014.05.006 24968370

[14] Campos-Vázquez MÁ Casamichana-Gómez D Suárez-Arrones L González-Jurado JA. Medium-sided games in soccer: physical and heart rate demands throughout successive working periods. J Hum Sport Exerc. (2017) 12(1):129–41. 10.14198/jhse.2017.121.11

[15] Abate Daga F Gollin M Daga AF. Manipulation of playing field’s length/width ratio and neutral players’ positioning: activity profile and motor behavior demands during positional possession soccer small sided games in young elite soccer players. Int J Sports Sci. (2016) 6:106–15. 10.5923/j.sports.20160603.07

[16] Couto BP Praça GM Gabbett TJ Luchesi MS Oliveira MP Sayers MGL. The influence of the field orientation on the representativeness of the positional dynamics in soccer small-sided games. Int J Sports Sci Coach. (2023) 19(4):1680–87.

[17] Casamichana D Bradley PS Castellano J. Influence of the varied pitch shape on soccer players physiological responses and time-motion characteristics during small-sided games. J Hum Kinet. (2018) 64(1):171–80. 10.1515/hukin-2017-0192 30429909 PMC6231353

[18] Weaving D Young D Riboli A Jones B Coratella G. The maximal intensity period: rationalising its use in team sports practice. Sports Med Open. (2022) 8(1):128. 10.1186/s40798-022-00519-7 36224479 PMC9556679

[19] Asian-Clemente J Rabano-Muñoz A Muñoz B Franco J Suarez-Arrones L. Can small-side games provide adequate high-speed training in professional soccer? Int J Sports Med. (2021) 42(6):523–8. 10.1055/a-1293-8471 33176385

[20] Clemente FM. The threats of small- sided soccer games: a discussion about their differences with the match external load demands and their variability levels. Strength Cond J. (2020) 42(3):100–5. 10.1519/SSC.0000000000000526

[21] de Dios-Álvarez V Castellano J Padrón-Cabo A Rey E. Do small-sided games prepare players for the worst-case scenarios of match play in elite young soccer players? Biol Sport. (2024) 41:95–106. 10.5114/biolsport.2024.127389 PMC10765451 38188112

[22] Oliva-Lozano JM Rojas-Valverde D Gómez-Carmona CD Fortes V Pino-Ortega J. Worst case scenario match analysis and contextual variables in professional soccer players: a longitudinal study. Biol Sport. (2020) 37(4):429–36. 10.5114/BIOLSPORT.2020.97067 33343077 PMC7725043

[23] Bortnik L Nir O Forbes N Alexander J Harper D Bruce-Low S Worst case scenarios in soccer training and competition: analysis of playing position, congested periods, and substitutes. Res Q Exerc Sport. (2023) 95:588–600. 10.1080/02701367.2023.2290265 38100605

[24] Casamichana D Castellano J Diaz AG Gabbett TJ Martin Garcia A. The most demanding passages of play in football competition: a comparison between halves. Biol Sport. (2019) 36(3):233–40. 10.5114/biolsport.2019.86005 31624417 PMC6786330

[25] Martin Garcia A Castellano J Diaz AG Cos F Casamichana D. Positional demands for various-sided games with goalkeepers according to the most demanding passages of match play in football. Biol Sport. (2019) 36(2):171–80. 10.5114/biolsport.2019.83507 31223195 PMC6561222

[26] Martín García A Casamichana D Díaz Gómez A Cos F Gabbett JT. Positional differences in the most demanding passages of play in football competition. J Sports Sci Med. (2018) 17(4):563–70. PMCID: . 30479524 PMC6243617

[27] Riboli A Esposito F Coratella G. Small-sided games in elite football: practical solutions to replicate the 4 min match-derived maximal intensities. J Strength Cond Res. (2023) 37(2):366–74. 10.1519/JSC.0000000000004249 35333202

[28] Cipriano R Pérez-Chao E Lago C Zong S Gómez MÁ. Frequency and intensity of maximal and submaximal demanding scenarios in U19 professional soccer players. J Sports Sci. (2024) 42(12):1112–9. 10.1080/02640414.2024.2384256 39058913

[29] Novak AR Impellizzeri FM Trivedi A Coutts AJ McCall A. Analysis of the worst-case scenarios in an elite football team: towards a better understanding and application. J Sports Sci. (2021) 39(16):1850–9. 10.1080/02640414.2021.1902138 33840362

[30] Oliva-Lozano JM Fortes V Muyor JM. The first, second, and third most demanding passages of play in professional soccer: a longitudinal study. Biol Sport. (2021) 38(2):165–74. 10.5114/biolsport.2020.97674 34079161 PMC8139346

[31] Caro E Campos-Vázquez MÁ Lapuente-Sagarra M Caparrós T. Analysis of professional soccer players in competitive match play based on submaximum intensity periods. PeerJ. (2022) 10:e13309. 10.7717/peerj.13309 35497181 PMC9053299

[32] Riboli A Esposito F Coratella G. The distribution of match activities relative to the maximal intensities in elite soccer players : implications for practice. Res Sports Med. (2021) 29:1–12. 10.1080/15438627.2021.1895788 33657944

[33] Caro E Lapuente-Sagarra M Caparrós T Pajón D Campos-Vázquez MÁ. Analysis of the submaximal intensity periods during the competitive microcycle in professional football players. Apunt Educ Fis Depor. (2024) 158:52–62. 10.5672/apunts.2014-0983.es.(2024/4).158.06

[34] Lobo-Triviño D García-Calvo T Polo-Tejada J Sanabria-Pino B López del Campo R Nevado-Garrosa F Analyzing positional and temporal variations in worst-case scenario demands in professional Spanish soccer. J Funct Morphol Kinesiol. (2025) 10(2):172. 10.3390/jfmk10020172 40407456 PMC12101334

[35] Illa J Fernandez D Reche X Carmona G Tarragó JR. Quantification of an elite futsal team’s microcycle external load by using the repetition of high and very high demanding scenarios. Front Psychol. (2020) 11(October):1–10. 10.3389/fpsyg.2020.577624 33178080 PMC7593252

[36] World Medical Association. World Medical Association declaration of Helsinki: ethical principles for medical research involving human subjects. JAMA. (2013) 310(20):2191–4. 10.1001/jama.2013.281053 24141714

[37] Jennings D Cormack S Coutts AJ Boyd LJ Aughey RJ. Variability of GPS units for measuring distance in team sport movements. Int J Sports Physiol Perform. (2010) 5(4):565–9. 10.1123/ijspp.5.4.565 21266740

[38] Beato M Coratella G Stiff A Dello Iacono A. The validity and between-unit variability of GNSS units (STATSports apex 10 and 18 Hz) for measuring distance and peak speed in team sports. Front Physiol. (2018) 9(SEP):1–8. 10.3389/fphys.2018.01288 30298015 PMC6161633

[39] Delves RIM Duthie GM Ball KA Aughey RJ. Applying common filtering processes to global navigation satellite system-derived acceleration during team sport locomotion. J Sports Sci. (2022) 40(10):1116–26. 10.1080/02640414.2022.2051332 35282785

[40] Illa J Fernandez D Tarragó JR Reche X. Most demanding passages in elite futsal: an isolated or a repeat situation? Apunt Educ Fis Depor. (2020) 142:80–4. 10.5672/apunts.2014-0983.es.(2020/4).142.10

[41] Yılmaz O Soylu Y. A comparative study of the effects of small-sided game formats on internal load and technical responses in soccer. Pamukkale J Sport Sci. (2024) 15:416–31. 10.54141/psbd.1467311

[42] Searle SR Speed FM Milliken GA. Population marginal means in the linear model: an alternative to least squares means. Am Stat. (1980) 34:216–21. 10.2307/2684063

[43] Santos FJ Verardi CEL de Moraes MG Filho P Macedo DM Figueiredo AG Effects of pitch size and goalkeeper participation on physical load measures during small-sided games in sub-elite professional soccer players. Appl Sci. (2021) 11(17):1–12. 10.3390/app11178024

[44] Riboli A Coratella G Rampichini S Cé E Esposito F. Area per player in small-sided games to replicate the external load and estimated physiological match demands in elite soccer players. PLoS One. (2020) 15:e0229194. 10.1371/journal.pone.0229194 32966305 PMC7510966

